# Knowledge, motivation, and attitudes of Hungarian family physicians toward pandemic influenza vaccination in the 2009/10 influenza season: questionnaire study

**DOI:** 10.3325/cmj.2011.52.134

**Published:** 2011-04

**Authors:** Imre Rurik, Zoltán Langmár, Hajnalka Márton, Eszter Kovács, Endre Szigethy, István Ilyés

**Affiliations:** 1 Department of Family and Occupational Medicine, Public Health Research Group of the Hungarian Academy of Science, Faculty of Public Health, Medical and Health Science Center, University of Debrecen, Debrecen, Hungary; 2 Second Department of Obstetrics and Gynecology, Semmelweis University, Budapest, Hungary; 3 Department of Preventive Medicine, Public Health Research Group of the Hungarian Academy of Science, Faculty of Public Health, Medical and Health Science Center, University of Debrecen, Debrecen, Hungary

## Abstract

**Aim:**

To evaluate the knowledge, motivation, and attitudes of Hungarian family physicians toward pandemic influenza vaccination in the 2009/10 influenza season.

**Method:**

A questionnaire with 20 questions was developed and sent to 232 family physicians in 3 largest Hungarian cities: Budapest, Debrecen, and Miskolc. The study was conducted in December 2009 and January 2010.

**Results:**

A hundred and ninety eight (85%) physicians answered the questionnaire adequately. Respondents believed that the influenza outbreak represented less of a threat to their practices than to Hungary or the world as a whole. They mostly agreed that vaccination was important and were frequently dissatisfied with the support from health authorities. The proportion of vaccinated patients ranged between 2% and 53%, without differences according to geographical region, age, sex, and duration of physicians’ employment in family practice. Physicians who were satisfied with the payment for procedures and underwent vaccination themselves were more active in vaccination.

**Conclusion:**

Health authorities should provide clear and evidence-based professional support to family physicians and should encourage them to get vaccinated against pandemic influenza, while insurance funds have to establish appropriate reimbursement system.

In June 2009, the World Health Organization (WHO) announced the global pandemic of influenza A/California 07/09 (H1N1). In Hungary, the media and the leading Hungarian medical journal reported on the emergence of a novel strain of swine-origin virus (1). The symptoms were usually mild and prevention was important, with an emphasis on the adherence to hygiene and vaccination that was recommended for everyone at risk of becoming ill or of transmitting the virus. It was not recommended for pregnant women in the first trimester, due to a lack of experience with the vaccine in this population. The pandemic had been predicted by infectologists years before (2,3).

The Hungarian government established the Committee for Pandemic, and family physicians were ordered to prepare weekly surveillance reports and lists of people who needed vaccinations. Posters on personal hygiene and behavior recommendations were displayed in public spaces (4) and at the end of the summer of 2009 thermo-gates were deployed at airports to detect incoming people with fever.

Vaccination was provided free of charge for health care staff, inpatients in hospitals and nursing homes, people over 60 years of age, patients with chronic pulmonary or heart diseases, children and adults who were assumed to be in danger of infection, police officers, workers of the public transport and services, and patients on long-term aspirin treatment (2).

Influenza AH1N1 variant arrived in Hungary in July 2009 (2,5). The Hungarian Fluval P (Omninvest, Pilisborosjenő, Hungary) vaccine was developed as a brand of the previously used Fluval H5N1 vaccine, containing thiomersal as preservative agent (6), which was added to other vaccines used in the USA as well (7). Clinical evaluation in Hungary started in August in line with the European Union regulations (8). After some modifications, the pharmaceutical authority allowed its use in adolescents and later in children.

During the initial weeks, there were logistical problems with vaccine distribution. The vaccine was delivered by the local offices of the Chief Health Officer. For those who were vaccinated free of charge, the Hungarian Health Insurance Fund offered HUF 200 (cca € 0.75) extra payments to family physicians per injection. For those who were not vaccinated free of charge, the price was HUF 1000-1500 (€ 4-6) per injection.

Through September and October 2009, lay press reported on a disagreement between experts on the safety and effectiveness of the vaccine. The most frequently discussed problem was the vaccination of pregnant women and children (1,3).

Many family physicians lacked confidence in the vaccine and asked the patients to sign that they assume the responsibility for any side effects or complications. This practice was supported by the Medical Chamber and other professional organizations, but was prohibited by the Minister of Health. One of the patient organizations pressed charges against the government (9). There were family physicians who recommended the vaccination and others who dissuaded their patients from getting vaccinated. The issue whether to start an antiviral treatment with neuraminidase inhibitors was also widely discussed (10,11).

For people who were vaccinated free of charge, a network of “vaccination-points” was organized by mid-November 2009 in the county offices of the health authority and in larger hospitals. At these points, the whole reimbursement was HUF 3000 ( ≈ € 11), covering the price of the vaccine and the procedure. However, some suspicions over the financial management of these points were raised (12).

Different opinions on the effectiveness of the vaccine were published in daily newspapers and media broadcasts. Some opposition politicians suggested that key government officials were among the owners of the vaccine manufacturer (13). Many of the opposition politicians refused to be vaccinated, while prominent government members, including the Minister of Health, underwent vaccination in a highly publicized manner.

In the mid-December, there were reports on deaths of mothers and their newborns attributable to H1N1. The position of the strongest opposition party toward vaccination then changed and the party opened its own “vaccination point” in the center of Budapest (14). One of the leading opposition politicians blamed the government for buying the cheaper vaccine (13). In October, 73% of the polled were against the vaccination and in November only 57% were against it, while 28% supported the vaccination and 15% hesitated. By mid-December, the ratio of vaccinated people was two times higher among the government supporters than among opposition supporters (13,15).

The aim of this study was to evaluate the knowledge, motivation, and attitudes of family physicians regarding vaccination in the 2009/10 pandemic influenza season.

## Methods

After discussions with family physicians and public health experts, a questionnaire was developed, with 20 questions in Hungarian language (web extra material)(web extra material 1) . There were 16 multiple-choice questions, 2 open-ended questions inquiring about general data on family practices and vaccination-related activities, and 2 open-ended questions on respondents’ personal experiences and recommendations for the future. For some questions, more than one answer was accepted. The questionnaires were distributed during continuing medical education courses and other meetings of family physicians in Budapest and other two largest Hungarian cities, Debrecen and Miskolc, between December 2009 and January 2010. Participating physicians worked in these and surrounding smaller cities and rural areas.

The questions, answers, and their distribution are presented in the order they appeared in the questionnaire. Results from the cities are reported together, except when significant differences were found between them.

The statistical analysis was performed by Stata 8.2 software (Statacorp LP, College Station, TX, USA). Fisher exact test, Mann-Whitney test, and Kruskal-Wallis equality of populations rank tests were used. The level of significance was set at *P* < 0.05.

## Results

Out of 232 distributed questionnaires, 198 were collected and analyzed (response rate: 85%). The mean time ± standard deviation duration of respondents’ employment in medical practice was 22.4 ± 12.1 years. Sixty one percent of respondents were male.

Physicians believed that the pandemic threat was greater for the whole world than for the Hungarian population or their own practice population (Table 1). Twenty of 30 physicians from Budapest (66.6%) considered vaccination as the most effective way of influenza prevention, as well as 89% of physicians from other two cities (103 of 116 in Debrecen and 46 of 52 in Miskolc) (*P* = 0.09, Fisher exact test). Thirty four (29.7%) physicians from Debrecen believed that pharmaceutical companies were behind the media campaign, which is significantly more than 2 physicians from Budapest (6.7%) (*P* = 0.039, Fisher exact test). Physicians believed that they were much better informed on the issues related to H1N1 vaccination than the general public (Table 2). Respondents reported that they more frequently received information on the pandemics from the media than from the Health Ministry and the Insurance Fund, with whom they were professionally contracted (Table 3). Regarding logistic problems experienced during the first weeks of the campaign, physicians criticized pharmacists for not ordering and selling the vaccine. Most of the respondents considered the paper-work associated with the vaccinations to be exaggerated and often superfluous. At the same time, there was general satisfaction with the payment received for the procedures (Table 4).

**Table 1:** Family physicians’ (n = 198) estimation of pandemic threat for different populations

Threat of pandemic influenza for the population in	No (%) of physicians who answered the question				
	extreme	serious	medium	moderate	none
the whole world	18 (9.1)	83 (41.9)	71 (35.9)	26 (13.1)	0
Hungary	11 (5.6)	74 (37.4)	76 (38.4)	37 (18.7)	0
your practice	5 (2.5)	55 (27.8)	82 (40.9)	51(25.8)	5(2.5)

**Table 2:** Family physicians’ (n = 198) opinion on preventive measures, media publicity, and how informed the general public and physicians are of influenza

Question	No. (%) of physicians who answered the question
What is the best way to prevent the pandemic influenza more effectively?*	
vaccination	170 (86.1)
closing borders	5 (2.5)
local quarantine	5 (2.5)
hygienic regulations	75 (38.0)
How proportional is the media publicity to the real danger? *	l

proportional	25 (12.6)
too big	134 (67.7)
prompted by the pharmaceutical companies	42 (21.2)
too small	5 (2.5)
How informed is the Hungarian population about the danger of influenza?	
sufficiently	25 (12.6)
partially	83 (41.9)
poorly	77 (38.9)
uninformed	13 (6.6)
How informed are the Hungarian family physicians’ about the danger of influenza?	
sufficiently	90 (45.4)
partially	77 (38.9)
poorly	31 (15.7)
uninformed	0

*More than one answer was possible.

**Table 3:** Family physicians’ (n = 198) opinions on the contribution of different institutions to the vaccination campaign*

Question	No. (%) of physicians who answered the question
From whom did the physicians get the most useful information?	
Ministry of Health	33 (16.7)
Chief Health Officer	152 (76.8)
Health Insurance Fund	25 (12.6)
media	74 (37.3)
Who should have provided more information to the population?	
Ministry of Health	124 (62.6)
Chief Health Officer	93 (47.0)
Health Insurance Fund	29 (14.7)
media	49 (24.8)
Who’s performance was worse than expected?	
Ministry of Health	108 (54.6)
Chief Health Officer	112 (56.6)
Primary care staff	48 (24.2)
pharmacists	6 (3.0)
Who should have gotten more tasks in vaccine administration?	
Ministry of Health	110 (55.6)
Chief Health Officer	99 (50.0)
Primary care staff	9 (4.6)
pharmacists	16 (8.1)

*For all the questions, more than one answer was possible.

**Table 4:** Family physicians’ (n = 198) opinion on the administration of vaccination, payment to physicians, and self-vaccination

Question	No. (%) of physicians who answered the question
Was the administration of vaccination:	
proportional	27 (13.6)
bureaucratic	116 (58.6)
too complicated	51 (25.8)
simple	4 (2.0)
Was the payment to physicians:	
proportional	79 (40.0)
correct	71 (35.9)
clearly determined	48 (24.2)
Self-vaccination against infections:	
pandemic	154 (77.8)	
seasonal	105 (53.0)	
pneumococcal	7 (3.5)	
other	1 (0.5)	
none	44 (22.2)	

On the scale from 1 (low) to 5 (high), the safety of the Hungarian vaccine was rated with 5 by 65 (36.2%) respondents, with 4 by 89 (45%), with 3 by 22 (11%), with 2 by 6 (3.2%), and with 1 by 6 (3.2%). On the same scale, the reliability of the Hungarian vaccine was rated with 5 by 90 (46%) respondents, with 4 by 70 (36%), with 3 by 24 (12%), with 2 by 4 (2.1%), and with 1 by 6 (3.1%).

Hundred and sixteen physicians (59%) registered only mild, transient adverse events in their patients following vaccination, and 79 (40%) did not register any adverse event. The mean percentage ± standard deviation of minor reactions was 11.0 ± 8.2 in Budapest, 2.3 ± 1.8 in Debrecen, and 1.5 ± 1.3 in Miskolc (*P* = 0.045, Kruskal-Wallis test). There were 11.0 ± 8.2% minor reactions in Budapest, which is higher than in both Debrecen (2.3 ± 1.8%) and Miskolc (1.5 ± 1.3%) (*P* = 0.045, Kruskal-Wallis test). A hundred and eighty eight (95%) physicians gave more than 100 injections during the process of immunization and 99 (50%) between 200 and 400. There was a wide gap between practices regarding the proportion of vaccinated people. This percentage varied between 2% and 53% of practice populations (median, 15%; inter-quartile range, 10-22%), without differences in geographical location.

The 143 physicians who were vaccinated themselves had a median of practice vaccination of 18% (inter-quartile range, 12-22%), while the 55 physicians who were not vaccinated had 13% (inter-quartile range, 8-20%) (*P* = 0.046, Mann-Whitney test). Physicians who considered the payment for the procedures proportional or correct were significantly more active in the vaccination than those who thought that it was only clearly determined (*P* = 0.017, Kruskal-Wallis test). There were no differences in sex of physicians and duration of employment in the practice between physicians in all 3 cities.

In the questionnaire, physicians had the opportunity to give comments or recommendation for the future. Seventy eight physicians recommended better organization by official bodies and giving more information to family physicians. Many of the respondents (69%) were upset by the politicians’ influence on the professional issues. Seventy-four percent thought that the media influence was too strong and that the media inaccurately reported on politicians’ and experts’ statements.

## Discussion

Hungarian physicians who believed in the effect of immunization and were satisfied with financial incentives were more active in the pandemic influenza vaccination campaign in the season 2009/2010. Physicians had no previous experiences in managing an epidemic and they had huge expectations from professional bodies and authorities, so initial logistic and organizational failures, accompanied by contradictive information arising from the political debate and media campaigns on the influenza, created uncertainty and confusion among them.

As far as patients are concerned, it seems that they preferred the guidance of their favorite politician to that of their health care professionals (13,15). Also, different levels of self-vaccination among physicians, as reported in our study, might have contributed to the lack of trust among patients, since they expect from health care professionals to be exemplary persons (11).

A limitation of our study is that it covered a small sample of family physicians, which has not been confirmed to be representative. Furthermore, possible changes in their vaccination-related activity during the campaign were not evaluated, although they may have been influenced by the general public atmosphere in Hungary in the winter of 2009/2010 (13-15). Also, they may have been influenced by acceptance of payments from the patients and from the Health Insurance Fund, but that issue was not assessed in our study.

There are other European countries in which problems with vaccination were reported. In Germany a “perfect chaos” was caused by different recommendations – politicians and soldiers got a presumably better type of vaccine than the largest part of the population (16). The indication was the same, but different schemes for injections and vaccines were provided by well-known multinational companies. Spanish experts were skeptical about the use of vaccination. They thought it to be irrational and based on fear-mongering rather than on “common sense and self control” (17). In the UK, the greatest problem, as perceived by the surveyed family physicians, were unclear, duplicate, and conflicting pieces of information (18).

Another important issue in Hungary was a professional disagreement on the immunization during pregnancy. In other countries, pregnant women were recommended seasonal influenza vaccination and identified as a priority group in the event of a pandemic. Vaccination in any trimester during the pandemic is supported by excess morbidity and mortality in the two previous influenza pandemics (19,20), as well as confirmed by recent WHO guidelines (21).

The viral or virion origin of vaccine and added preservative agents were very different worldwide. Because of the imminent pandemic threat there were countries, even in Europe, where detailed clinical tests were not carried out with the new vaccine. Only limited data on safety and immunogenicity of influenza A/H1N1 vaccines were available when EU member states started using them (22).

The present pandemic vaccine used in Hungary proved safe and immunogenic in healthy adults and the elderly. It could be safely co-administered with the 2009/10 seasonal influenza vaccine (8). Physicians using this vaccine in our study had similar experiences.

Not only physicians, but also the patients needed more information before making a decision about vaccination. The Hungarian Health Insurance Fund did not provide a informational leaflet for patients, while the informational leaflet of the National Health Services had already been available in the summer of 2009 (23).

At the end of March, it became clear that the epidemic had not been as serious as anticipated. The obligation to send weekly a report to the Health Officer was abolished and “vaccination points” were closed. The government ordered 6 million vaccines for a population of 10 million. Four million were offered for free and 2 million were reimbursed by the patients. Large amount of ordered vaccine was not used. According to the recent available data, 3.34 million doses of vaccine were used, 1.3 million were given to the high-risk population for free, and 129 000 to health staff. A hundred and twenty nine fatalities were reported as a consequence of the pandemic and 1 as a consequence of seasonal influenza (24). The H1N1-related mortality in Hungary was among the highest in Europe, but this could be explained by the precise laboratory and pathological examination after each death (25,26).

In the past few months, information about the relation between pharmaceutical companies, WHO experts, and vaccination advisors has been published (27). Many of the surveyed Hungarian physicians had similar suspicion.

In other countries, discussions and arguments were mainly professional, typically not influenced by politicians (16-18). In Hungary, however, poor or inappropriate communication with the government, animosity between political parties, logistic and organizational failures at the outset of the campaign, and the lack of confidence in the governmental institutions could have caused the confusion among physicians (13). These factors were combined with the hysteria initiated and maintained by the media (28).

Health authorities should provide clear and evidence-based professional support for family physicians and should encourage self-vaccination of physicians, while insurance funds have to establish appropriate reimbursement system. Authors hope that their experiences can prepare other primary care systems for any infectious threat in the future.
