# Supplementary material for: Knowledge, motivation, and attitudes of Hungarian family physicians toward pandemic influenza vaccination in the 2009/10 influenza season: questionnaire study
Source: Croat Med J. 2011 Apr;52(2):134–40. doi: 10.3325/cmj.2011.52.134 (PMC3081211; doi:10.3325/cmj.2011.52.134)
Supplement: Supplementary Table 1 [file CroatMedJ_52_s003.pdf]

Tisztelt Kollega! 10.jan.14.

Tanszékünk tudományos munkájához nagyon fontos, hogy megismerjük háziorvos kollegáink véleményét egyes szakmai kérdésekről.

Várjuk véleményét válaszait az egyik legfontosabb aktuális témáról, a pandemiás (H_1N_1) influenzáról, illetve a védőoltásról.

A következő kérdésekre adott egyértelmű válasza (aláhúzással vagy bekarikázással), sokat segít nekünk, és ezt megköszönjük !

DE OEC Családorvosi és Foglalkozás-egészségügyi Tanszék

Dear Colleague!

Our department is open for the opinion of our colleagues and for common thinking about professional issues.

We are asking your opinion about one of the most relevant and important issue, the H1N1 pandemic and the vaccination against it.

We ask you to answer the following questions with unambiguous way, underlying or circle around the most preferred answer options.

It would be a great help for us and thanks for it!

Department of Family and Occupational Medicine

ÖN SZERINT,

1./ Mennyire jelent veszélyt a **Föld lakossága** számra a pandemiás influenza?

nagyon súlyos - elég súlyos - közepes - mérsékelt – semmilyen

According to your opinion,

1.How dangerous is the pandemic flu for the whole **World**?

extreme- serious-medium-moderate-none

2./ Mennyire jelent veszélyt a **magyar lakosság** számára?

nagyon súlyos - elég súlyos - közepes - mérsékelt – semmilyen

2. How dangerous is it for the population of **Hungary**?

extreme - serious -medium- moderate - none

3./ Mennyire jelent veszély az **Ön körzeté**nek lakosságára?

nagyon súlyos - elég súlyos - közepes - mérsékelt – semmilyen

3. How dangerous is it for your **own practice** population?

extreme - serious -medium- moderate - none

4./ Hogyan lehet még eredményesebben védekezni a megbetegedések ellen?

védőoltással – teljes határzárral – lokális karanténnal – higiénés korlátozásokkal – sehogy

4. What is the best way to prevent pandemic more effectively?

with vaccination-closing borders-local quarantine-hygienic regulations-no way

5./ A sajtóban kapott publicitás arányban áll-e a valós veszéllyel?

arányban áll – a sajtó túlzottan foglalkozik vele – gyógyszercégek mozgatják a médiakampányt –még így is túl keveset beszélnek róla

5./ How proportional is the media publicity to the real danger?

in proportion- too much focus in media-publicity is moved by pharmaceutical companies- media publicity is too small

6./ Mennyire informált a magyar lakosság a veszélyről?
megfelelően tájékoztatott- részben informált – hiányosan informált – tájékozatlan

6. How informed is the Hungarian population about the danger of flu?
sufficiently-partially-poorly-uninformed

7./ Mennyire informáltak a magyar háziorvosok?
megfelelően tájékozottak- részben informáltak – hiányosan informáltak – tájékozatlanok

7. How informed are the Hungarian family physicians about the danger of flu?
sufficiently-partially-poorly-uninformed

8./ Honnan kapták a legtöbb információt a háziorvosok?
médiából- Eü.Min.- OEP – ÁNTSz - politikai pártok – betegek

8. From whom get family physicians the most useful information?
media-Ministry of Health- Health Insurance Fund-political parties-patients

9./A forgalmazott magyar védőoltás mennyire hatásos? (értékelje 1→5 között) ____

9. How effective is the Hungarian vaccine? (please rate it from 1 to 5)

10./ A forgalomban lévő magyar védőoltás mennyire megbízható? (1→5)

10./ How reliable is the Hungarian vaccine? (please rate it from 1 to 5)

11./ Kinek lett volna több feladata a védőoltás kampány lebonyolításában?
Eü.Min. – ÁNTSz – gyógyszerészek – háziorvosok – védőnők

11. Who should have more task in the vaccination campaign and logistic?
Ministry of Health –health officers-pharmacists-family physicians-health visitors

12./ Kinek lett volna több feladata a lakosság tájékoztatásában?
média - politikai pártok – OEP - Eü.Min. - ÁNTSz - betegszervezetek – iskolák- orvosok

12. Who should have provided more information for the population?
media-political parties-Health Insurance Fund-health officers-patient organizations-schools-physicians

13./ Kinek lett volna több feladata az oltóanyag ellátás lebonyolításában?
Eü.Min. – ÁNTSz – gyógyszerészek – háziorvosok – védőnők

13. Who should have more tasks in the vaccine logistic?
Ministry of Health-health officers-pharmacists-family physicians-health visitors

14./ Az oltások adminisztrációjára mi jellemző?
megfelelően arányos – bürokratikus - túlzott- egyszerű

14. How was the administration of vaccination?
proportional-burocratic-too complicated-simple

15./ Az oltások beadása után milyen szövődményeket látott és milyen arányban?
egyáltalán nem – egyszerű oltási reakciót – egyéb:………..
adatok: …. beadott oltás közül ……. darabot

15.What complications have you experienced, and in which ratio?
nothing-simple reactions-other………(please specify)

data: from………number of vaccination……….number of cases

16./ Az Ön praxisában eddig kb. mennyi vakcináció történt?
…… fő, ami a praxislétszám (. . . . fő) %-a

16. Approximately how many vaccinations were performed in your practice?
by …………persons, which is ……..% of the practice population of ………persons

17./ A háziorvosok díjazása az oltásokkal kapcsolatban:
megfelelő, korrekt- arányos a feladattal- jogilag rendezett és egyértelmű

17. How was the payment of physicians for vaccination?
adequate, correct – in direct proportion to task – legally unambiguous, clear

18./ Ön milyen oltást kapott az idén?
Pandemiás influenza, szezonális influenza – pneumococcus – semmit- egyéb, mégpedig:

18. You are vaccinated in this season against ……
pandemic flu, seasonal flu, pneumococcus-none-other (specify)

19/ Milyen tanulságot jelent az idei oltási kampány az Ön számára?

19. What did you learn during this vaccination campaign?

……………………………………………………………………………

……………………………………………………………………………

……………………………………………………………………………

20./ Milyen tanácsot /ajánlást adna és melyik szervezetnek/hivatalnak?

20. What would be your recommendation and for which bodies, institutions?

……………………………………………………………………………

……………………………………………………………………………

……………………………………………………………………………

……………………………………………………………………………

Kitől várt volna több segítséget a problémák megoldásában?
Eü. Min. ÁNTSZ, Egyetemek, Szakirodalom, Orvosi Kamara, egyéb:

From whom did you expect more help? Ministry of Health, health officers, Universities, scientific literature, Medical Chamber, other

…………………………………………………………………………….
